# Supplementary material for: A Bibliometric Review of the Keap1/Nrf2 Pathway and its Related Antioxidant Compounds
Source: Antioxidants (Basel). 2019 Sep 1;8(9):353. doi: 10.3390/antiox8090353 (PMC6769514; doi:10.3390/antiox8090353)
Supplement: Supplementary file 1 [file antioxidants-08-00353-s001.zip › Figure S3.pdf]

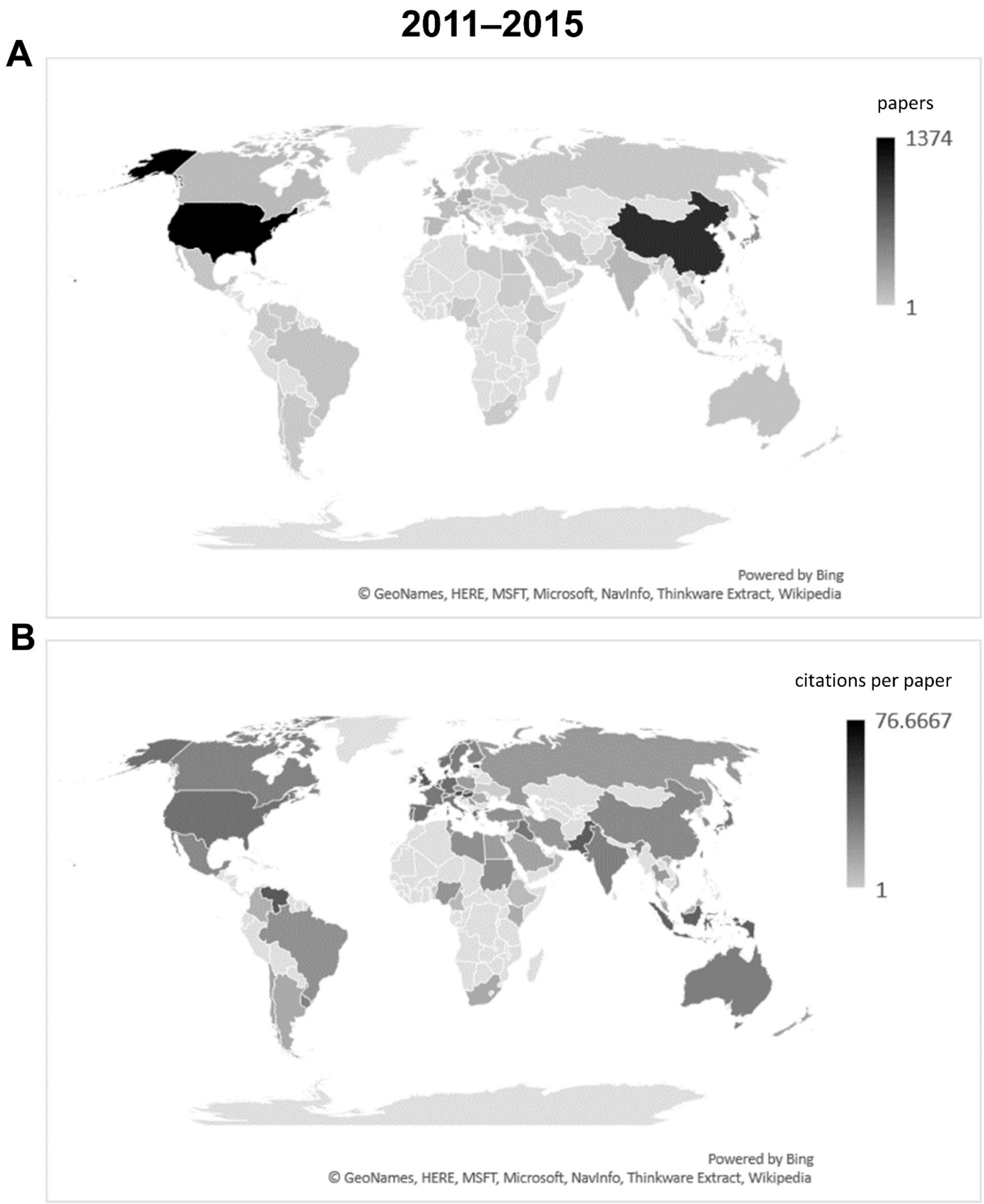

Figure S3. World map depicting the number of Nrf2-related papers by each country in the period 2011–2015 and the respective citations received by these papers. Each paper may be counted by more than one country (international collaboration). **A. Number of papers by country**. The darker the color of

a country, the higher the number of Nrf2-related papers published. **B. Averaged citations per Nrf2-related paper per country.** The darker the color of a country, the higher the number of citations per Nrf2-related paper. Countries with at least 1 publication are depicted. Note that “England”, “Scotland”, “Wales” and “Northern Ireland” records from the “Web of Science” were combined into “UK”.
