# Supplementary material for: A Bibliometric Review of the Keap1/Nrf2 Pathway and its Related Antioxidant Compounds
Source: Antioxidants (Basel). 2019 Sep 1;8(9):353. doi: 10.3390/antiox8090353 (PMC6769514; doi:10.3390/antiox8090353)
Supplement: Supplementary file 1 [file antioxidants-08-00353-s001.zip › Table S1.docx]

**Table S1. Terms occurrences and averaged citations from 1990-2005**

| **terms** | **occurrences** | **averaged citations** |
| --- | --- | --- |
| oxidative stress | 187 | 172.8 |
| antioxidant response element | 158 | 193.5 |
| induction | 130 | 181.9 |
| expression | 90 | 205.3 |
| heme oxygenase-1 | 73 | 179.5 |
| gene-expression | 66 | 190.6 |
| activation | 60 | 170.6 |
| transcription factor | 57 | 198.8 |
| protein | 41 | 181.0 |
| subunit gene-expression | 41 | 214.2 |
| ya subunit gene | 40 | 237.3 |
| gamma-glutamylcysteine synthetase | 35 | 237.9 |
| glutathione s-transferase | 35 | 249.8 |
| nqo1 | 34 | 219.8 |
| glutathione | 32 | 134.4 |
| identification | 28 | 172.9 |
| phenolic antioxidants | 25 | 206.4 |
| sulfhydryl-groups | 23 | 239.5 |
| leucine zipper protein | 22 | 250.0 |
| cells | 21 | 97.5 |
| chemoprevention | 21 | 177.9 |
| maf | 21 | 271.2 |
| cloning | 20 | 248.0 |
| enzymes | 20 | 166.5 |
| gene | 20 | 195.2 |
| nf-kappa-b | 18 | 179.8 |
| apoptosis | 17 | 109.4 |
| element | 17 | 128.1 |
| mice | 17 | 215.4 |
| planar aromatic-compounds | 17 | 188.9 |
| antioxidant | 16 | 123.1 |
| binding | 15 | 140.1 |
| phosphatidylinositol 3-kinase | 15 | 155.5 |
| protein-kinase-c | 15 | 173.1 |
| carcinogenesis | 13 | 164.2 |
| family | 13 | 145.8 |
| human neuroblastoma-cells | 13 | 236.7 |
| mechanism | 13 | 265.4 |
| oligonucleotide microarray | 13 | 172.8 |
| rat-liver | 13 | 166.2 |
| transcription | 13 | 144.4 |
| butylated hydroxyanisole | 12 | 339.1 |
| cul3-based e3 ligase | 12 | 150.8 |
| degradation | 12 | 236.6 |
| endothelial-cells | 12 | 160.1 |
| in-vitro | 12 | 183.3 |
| inducers | 12 | 218.7 |
| functional-characterization | 11 | 144.5 |
| lipid-peroxidation | 11 | 89.4 |
| proteasomal degradation | 11 | 217.8 |
| c-fos | 10 | 118.4 |
| controlling inducible expression | 10 | 169.1 |
| liver | 10 | 72.2 |
| messenger-rna | 10 | 163.5 |
| mutant mice | 10 | 214.5 |
| cancer | 9 | 230.3 |
| carbon-monoxide | 9 | 156.6 |
| dna-binding | 9 | 246.4 |
| free radicals | 9 | 107.9 |
| keap1 | 9 | 342.6 |
| mediated rgsta2 induction | 9 | 111.7 |
| naphthoflavone-induced expression | 9 | 225.8 |
| tert-butylhydroquinone | 9 | 180.8 |
| consensus sequence | 8 | 123.9 |
| epithelial-cells | 8 | 191.8 |
| in-vivo | 8 | 273.0 |
| locus-control region | 8 | 150.0 |
| nitric-oxide synthase | 8 | 51.8 |
| pathway | 8 | 74.3 |
| sulforaphane | 8 | 197.5 |
| superoxide-dismutase | 8 | 182.4 |
| transgenic mice | 8 | 210.4 |
| electrophile | 7 | 397.9 |
| family proteins | 7 | 306.3 |
| fos | 7 | 169.6 |
| heavy-metals | 7 | 231.9 |
| jun | 7 | 256.7 |
| mammalian-cells | 7 | 261.1 |
| phase-2 enzymes | 7 | 161.6 |
| regulatory subunit gene | 7 | 173.1 |
| transcriptional regulation | 7 | 53.1 |
| ubiquitin-proteasome pathway | 7 | 284.9 |
| activated protein-kinases | 6 | 179.5 |
| alzheimers-disease | 6 | 51.7 |
| c-jun | 6 | 185.2 |
| cell-survival | 6 | 243.0 |
| conferring protection | 6 | 136.7 |
| detoxifying enzymes | 6 | 172.8 |
| dominant control region | 6 | 336.8 |
| drug-metabolizing-enzymes | 6 | 258.8 |
| element-mediated expression | 6 | 139.2 |
| inflammation | 6 | 128.2 |
| n-terminal kinase | 6 | 183.3 |
| phosphorylation | 6 | 130.8 |
| proteasome | 6 | 153.8 |
| protection | 6 | 172.5 |
| republic-of-china | 6 | 60.0 |
| 15-deoxy-delta(12,14)-prostaglandin j(2) | 5 | 199.8 |
| aflatoxin b-1 | 5 | 297.8 |
| ap-1 | 5 | 130.8 |
| aryl-hydrocarbon receptor | 5 | 179.6 |
| basal level | 5 | 272.6 |
| binding-site | 5 | 330.0 |
| bzip transcription factor | 5 | 182.0 |
| gene expression | 5 | 148.8 |
| heavy subunit gene | 5 | 42.6 |
| hepatoma-cells | 5 | 271.2 |
| inhibition | 5 | 301.8 |
| lung | 5 | 141.8 |
| macrophages | 5 | 130.8 |
| mapk | 5 | 154.8 |
| nf-e2 | 5 | 139.8 |
| nitric-oxide | 5 | 173.4 |
| nrf1 | 5 | 63.0 |
| nrf2-keap1 pathway | 5 | 103.0 |
| oltipraz | 5 | 276.6 |
| parkinsons-disease | 5 | 120.2 |
| phenethyl isothiocyanate | 5 | 166.0 |
| rat | 5 | 77.6 |
| redox regulation | 5 | 88.8 |
| regulatory mechanisms | 5 | 53.4 |
| small maf | 5 | 52.4 |
| tumor-necrosis-factor | 5 | 78.8 |
