# Supplementary material for: A Bibliometric Review of the Keap1/Nrf2 Pathway and its Related Antioxidant Compounds
Source: Antioxidants (Basel). 2019 Sep 1;8(9):353. doi: 10.3390/antiox8090353 (PMC6769514; doi:10.3390/antiox8090353)
Supplement: Supplementary file 1 [file antioxidants-08-00353-s001.zip › Table S10.docx]

**Table S10. Occurrences of natural compounds in Nrf2 publications from 1990–2019**

|  | **occurrences** | **averaged citations** |
| --- | --- | --- |
| sulforaphane | 337 | 29.3 |
| curcumin | 147 | 28.5 |
| resveratrol | 146 | 26.6 |
| flavonoids | 125 | 21.3 |
| quercetin | 118 | 19.6 |
| vitamin e | 86 | 18.1 |
| phytochemicals | 85 | 24.4 |
| tea polyphenols | 76 | 41.8 |
| phenolic-compounds | 66 | 87.5 |
| carnosic acid | 63 | 18.9 |
| isothiocyanate | 60 | 32.2 |
| phenethyl isothiocyanate | 43 | 71.6 |
| vitamin-c | 40 | 15.8 |
| anthocyanin | 38 | 16.8 |
| luteolin | 37 | 24.1 |
| oleanolic acid | 37 | 23.5 |
| caffeic acid phenethyl ester | 32 | 33.2 |
| lycopene | 29 | 18.8 |
| astaxanthin | 27 | 16.4 |
| berberine | 27 | 17.0 |
| coffee | 26 | 23.1 |
| triterpenoids | 26 | 22.8 |
| aflatoxin b-1 | 23 | 77.8 |
| genistein | 23 | 21.5 |
| baicalein | 20 | 18.5 |
| epigallocatechin gallate | 18 | 36.8 |
| ferulic acid | 18 | 21.2 |
| ellagic acid | 17 | 17.8 |
| puerarin | 17 | 17.6 |
| carotenoids | 16 | 25.3 |
| naringenin | 16 | 16.4 |
| rutin | 16 | 9.9 |
| silymarin | 16 | 15.6 |
| tanshinone iia | 16 | 10.6 |
| dietary polyphenols | 15 | 32.9 |
| indole-3-carbinol | 15 | 13.4 |
| kaempferol | 15 | 13.0 |
| rosmarinic acid | 15 | 9.7 |
| baicalin | 14 | 6.6 |
| beta-carotene | 14 | 31.1 |
| epigallocatechin-3-gallate | 14 | 21.7 |
| fisetin | 14 | 13.9 |
| gastrodin | 14 | 15.9 |
| isoliquiritigenin | 14 | 16.4 |
| essential oil | 13 | 6.9 |
| gallic acid | 13 | 10.6 |
| apigenin | 12 | 14.4 |
| brusatol | 12 | 10.5 |
| hesperidin | 12 | 11.6 |
| isothiocyanate sulforaphane | 12 | 36.0 |
| licorice | 12 | 19.3 |
| allicin | 11 | 13.6 |
| cinnamaldehyde | 11 | 21.7 |
| fish-oil | 11 | 28.6 |
| l-carnitine | 11 | 6.6 |
| lutein | 11 | 8.0 |
| ochratoxin a | 11 | 35.2 |
| phenolic-acids | 11 | 9.5 |
| quinone | 11 | 25.7 |
| (-)-epigallocatechin-3-gallate | 10 | 28.9 |
| chalcone | 10 | 25.4 |
| diallyl sulfide | 10 | 46.1 |
| diallyl trisulfide | 10 | 40.6 |
| flavonoid fisetin | 10 | 26.6 |
| red wine | 10 | 52.7 |
| trans-resveratrol | 10 | 47.1 |
| vitamin-a supplementation | 10 | 12.1 |
