# Supplementary material for: A Bibliometric Review of the Keap1/Nrf2 Pathway and its Related Antioxidant Compounds
Source: Antioxidants (Basel). 2019 Sep 1;8(9):353. doi: 10.3390/antiox8090353 (PMC6769514; doi:10.3390/antiox8090353)
Supplement: Supplementary file 1 [file antioxidants-08-00353-s001.zip › Table S11.docx]

**Table S11. Journals that have published at least 20 Nrf2-related papers from 1990-2019**

| **Journal** | **Papers #** |
| --- | --- |
| FREE RADICAL BIOLOGY AND MEDICINE | 445 |
| PLOS ONE | 366 |
| JOURNAL OF BIOLOGICAL CHEMISTRY | 228 |
| OXIDATIVE MEDICINE AND CELLULAR LONGEVITY | 222 |
| BIOCHEMICAL AND BIOPHYSICAL RESEARCH COMMUNICATIONS | 217 |
| SCIENTIFIC REPORTS | 209 |
| TOXICOLOGY AND APPLIED PHARMACOLOGY | 202 |
| BIOMEDICINE PHARMACOTHERAPY | 150 |
| FOOD AND CHEMICAL TOXICOLOGY | 141 |
| REDOX BIOLOGY | 141 |
| ONCOTARGET | 134 |
| ANTIOXIDANTS REDOX SIGNALING | 111 |
| INTERNATIONAL JOURNAL OF MOLECULAR SCIENCES | 111 |
| INTERNATIONAL IMMUNOPHARMACOLOGY | 109 |
| EUROPEAN JOURNAL OF PHARMACOLOGY | 101 |
| JOURNAL OF AGRICULTURAL AND FOOD CHEMISTRY | 94 |
| BIOCHEMICAL PHARMACOLOGY | 92 |
| MOLECULAR MEDICINE REPORTS | 92 |
| CHEMICO BIOLOGICAL INTERACTIONS | 89 |
| TOXICOLOGICAL SCIENCES | 88 |
| FREE RADICAL RESEARCH | 87 |
| JOURNAL OF FUNCTIONAL FOODS | 85 |
| TOXICOLOGY LETTERS | 83 |
| PROCEEDINGS OF THE NATIONAL ACADEMY OF SCIENCES OF THE UNITED STATES OF AMERICA | 81 |
| INTERNATIONAL JOURNAL OF MOLECULAR MEDICINE | 79 |
| FOOD FUNCTION | 75 |
| CHEMICAL RESEARCH IN TOXICOLOGY | 72 |
| MOLECULAR AND CELLULAR BIOLOGY | 72 |
| FISH SHELLFISH IMMUNOLOGY | 69 |
| FRONTIERS IN PHARMACOLOGY | 68 |
| LIFE SCIENCES | 68 |
| JOURNAL OF NUTRITIONAL BIOCHEMISTRY | 66 |
| NEUROCHEMICAL RESEARCH | 65 |
| MOLECULES | 64 |
| JOURNAL OF ETHNOPHARMACOLOGY | 62 |
| CELLULAR PHYSIOLOGY AND BIOCHEMISTRY | 61 |
| MOLECULAR NUTRITION FOOD RESEARCH | 61 |
| MOLECULAR NEUROBIOLOGY | 59 |
| TOXICOLOGY IN VITRO | 59 |
| CANCER RESEARCH | 56 |
| EVIDENCE BASED COMPLEMENTARY AND ALTERNATIVE MEDICINE | 56 |
| JOURNAL OF CELLULAR BIOCHEMISTRY | 54 |
| CELL DEATH DISEASE | 52 |
| PHYTOMEDICINE | 50 |
| INTERNATIONAL JOURNAL OF CLINICAL AND EXPERIMENTAL MEDICINE | 48 |
| NUTRIENTS | 47 |
| TOXICOLOGY | 46 |
| CARCINOGENESIS | 45 |
| NEUROCHEMISTRY INTERNATIONAL | 45 |
| INTERNATIONAL JOURNAL OF CLINICAL AND EXPERIMENTAL PATHOLOGY | 44 |
| MOLECULAR AND CELLULAR BIOCHEMISTRY | 44 |
| ARCHIVES OF TOXICOLOGY | 43 |
| BRAIN RESEARCH | 43 |
| JOURNAL OF CELLULAR PHYSIOLOGY | 43 |
| JOURNAL OF NEUROCHEMISTRY | 43 |
| RSC ADVANCES | 42 |
| ARCHIVES OF BIOCHEMISTRY AND BIOPHYSICS | 41 |
| ENVIRONMENTAL TOXICOLOGY AND PHARMACOLOGY | 41 |
| INVESTIGATIVE OPHTHALMOLOGY VISUAL SCIENCE | 40 |
| ONCOGENE | 40 |
| EXPERIMENTAL AND THERAPEUTIC MEDICINE | 39 |
| JOURNAL OF CELLULAR AND MOLECULAR MEDICINE | 39 |
| BIOLOGICAL PHARMACEUTICAL BULLETIN | 38 |
| AMERICAN JOURNAL OF RESPIRATORY CELL AND MOLECULAR BIOLOGY | 37 |
| NATURE COMMUNICATIONS | 37 |
| BIOMED RESEARCH INTERNATIONAL | 36 |
| BRITISH JOURNAL OF PHARMACOLOGY | 34 |
| ENVIRONMENTAL TOXICOLOGY | 34 |
| MOLECULAR PHARMACOLOGY | 34 |
| ACTA PHARMACOLOGICA SINICA | 32 |
| BIOCHEMICAL JOURNAL | 32 |
| BMC COMPLEMENTARY AND ALTERNATIVE MEDICINE | 32 |
| FASEB JOURNAL | 32 |
| INFLAMMATION | 32 |
| JOURNAL OF IMMUNOLOGY | 32 |
| JOURNAL OF MEDICINAL CHEMISTRY | 32 |
| NEUROSCIENCE LETTERS | 32 |
| DIABETES | 31 |
| DRUG METABOLISM AND DISPOSITION | 31 |
| JOURNAL OF NEUROINFLAMMATION | 31 |
| DRUG DESIGN DEVELOPMENT AND THERAPY | 29 |
| JOURNAL OF BIOCHEMICAL AND MOLECULAR TOXICOLOGY | 29 |
| PHYTOTHERAPY RESEARCH | 28 |
| BIOCHIMICA ET BIOPHYSICA ACTA MOLECULAR BASIS OF DISEASE | 26 |
| JOURNAL OF MEDICINAL FOOD | 26 |
| JOURNAL OF MOLECULAR NEUROSCIENCE | 26 |
| BIOCHEMICAL SOCIETY TRANSACTIONS | 25 |
| EUROPEAN REVIEW FOR MEDICAL AND PHARMACOLOGICAL SCIENCES | 25 |
| FEBS LETTERS | 25 |
| HEPATOLOGY | 25 |
| JOURNAL OF PHARMACOLOGY AND EXPERIMENTAL THERAPEUTICS | 25 |
| MARINE DRUGS | 25 |
| NEUROSCIENCE | 25 |
| AMERICAN JOURNAL OF PHYSIOLOGY LUNG CELLULAR AND MOLECULAR PHYSIOLOGY | 24 |
| AMERICAN JOURNAL OF TRANSLATIONAL RESEARCH | 24 |
| BIOCHIMICA ET BIOPHYSICA ACTA GENERAL SUBJECTS | 24 |
| EXPERIMENTAL CELL RESEARCH | 24 |
| HUMAN MOLECULAR GENETICS | 24 |
| MEDICAL SCIENCE MONITOR | 24 |
| ONCOLOGY LETTERS | 24 |
| PHARMACOLOGICAL RESEARCH | 24 |
| CELL REPORTS | 23 |
| CHEMOSPHERE | 23 |
| FRONTIERS IN IMMUNOLOGY | 23 |
| ONCOLOGY REPORTS | 23 |
| AMERICAN JOURNAL OF CHINESE MEDICINE | 22 |
| CANCER PREVENTION RESEARCH | 22 |
| NEUROTOXICOLOGY | 22 |
| AQUATIC TOXICOLOGY | 21 |
| JOURNAL OF NATURAL PRODUCTS | 21 |
| MEDIATORS OF INFLAMMATION | 21 |
| AGING CELL | 20 |
| BIOORGANIC MEDICINAL CHEMISTRY | 20 |
| CANCER LETTERS | 20 |
| FOOD CHEMISTRY | 20 |
| GENES TO CELLS | 20 |
| INTERNATIONAL JOURNAL OF BIOCHEMISTRY CELL BIOLOGY | 20 |
| INTERNATIONAL JOURNAL OF BIOLOGICAL MACROMOLECULES | 20 |
| NEUROTOXICITY RESEARCH | 20 |
