# Supplementary material for: A Bibliometric Review of the Keap1/Nrf2 Pathway and its Related Antioxidant Compounds
Source: Antioxidants (Basel). 2019 Sep 1;8(9):353. doi: 10.3390/antiox8090353 (PMC6769514; doi:10.3390/antiox8090353)
Supplement: Supplementary file 1 [file antioxidants-08-00353-s001.zip › Table S2.docx]

**Table S2. Terms occurrences and averaged citations from 2006–2010**

| **terms** | o**ccurrences** | a**veraged citations** |
| --- | --- | --- |
| oxidative stress | 676 | 73.0 |
| gene-expression | 338 | 71.4 |
| induction | 296 | 60.6 |
| heme oxygenase-1 | 288 | 64.4 |
| antioxidant response element | 275 | 75.0 |
| activation | 221 | 65.2 |
| expression | 193 | 53.8 |
| nf-kappa b | 182 | 68.7 |
| pathway | 153 | 70.0 |
| transcription factor | 150 | 65.7 |
| keap1 | 125 | 81.5 |
| glutathione | 123 | 55.6 |
| apoptosis | 112 | 51.0 |
| antioxidant | 106 | 66.6 |
| mechanism | 101 | 71.7 |
| cells | 99 | 64.3 |
| mice | 97 | 74.1 |
| reactive oxygen species | 93 | 61.1 |
| gene | 91 | 63.3 |
| in-vivo | 91 | 84.3 |
| in-vitro | 81 | 51.3 |
| inflammation | 75 | 62.6 |
| protein | 73 | 116.8 |
| chemoprevention | 70 | 64.7 |
| response element | 68 | 71.3 |
| glutathione-s-transferase | 65 | 54.7 |
| cul3-based e3 ligase | 64 | 161.6 |
| endothelial-cells | 63 | 64.0 |
| cancer | 59 | 76.2 |
| epithelial-cells | 56 | 58.7 |
| lipid-peroxidation | 55 | 71.1 |
| identification | 54 | 73.4 |
| sulforaphane | 53 | 65.3 |
| stress | 51 | 63.5 |
| protein-kinase-c | 50 | 94.4 |
| rat | 50 | 42.7 |
| free radicals | 48 | 62.5 |
| protection | 46 | 57.8 |
| gamma-glutamylcysteine synthetase | 45 | 71.3 |
| nitric-oxide | 44 | 59.9 |
| nqo1 | 41 | 71.3 |
| carbon-monoxide | 38 | 59.4 |
| carcinogenesis | 38 | 82.3 |
| enzymes | 38 | 48.7 |
| nitric-oxide synthase | 36 | 63.3 |
| subunit gene | 35 | 79.5 |
| liver | 34 | 57.3 |
| rat-liver | 34 | 40.0 |
| transcription | 34 | 63.0 |
| neuroprotection | 33 | 77.3 |
| element | 32 | 55.5 |
| hydrogen-peroxide | 32 | 64.0 |
| superoxide-dismutase | 32 | 70.0 |
| lung-cancer | 31 | 92.4 |
| degradation | 30 | 97.0 |
| inhibition | 30 | 62.4 |
| toxicity | 30 | 36.5 |
| proteasomal degradation | 29 | 166.1 |
| macrophages | 28 | 60.0 |
| alzheimers-disease | 27 | 59.3 |
| aryl-hydrocarbon receptor | 27 | 50.1 |
| enhances susceptibility | 27 | 124.7 |
| parkinsons-disease | 27 | 60.0 |
| phosphatidylinositol 3-kinase | 26 | 55.2 |
| adaptive response | 25 | 75.2 |
| dna-damage | 25 | 48.2 |
| messenger-rna | 25 | 62.1 |
| oligonucleotide microarray | 25 | 126.0 |
| smooth-muscle-cells | 25 | 53.1 |
| transcriptional regulation | 25 | 43.0 |
| cell-death | 24 | 77.5 |
| disease | 24 | 74.7 |
| drug-metabolizing-enzymes | 24 | 56.0 |
| human neuroblastoma-cells | 24 | 72.9 |
| brain | 23 | 80.0 |
| ubiquitination | 23 | 105.5 |
| binding | 22 | 66.0 |
| cell-survival | 22 | 76.2 |
| consensus sequence | 22 | 50.4 |
| injury | 21 | 35.6 |
| thioredoxin | 21 | 34.9 |
| atherosclerosis | 20 | 83.0 |
| obstructive pulmonary-disease | 20 | 107.5 |
| substrate adapter protein | 20 | 100.2 |
