# Supplementary material for: A Bibliometric Review of the Keap1/Nrf2 Pathway and its Related Antioxidant Compounds
Source: Antioxidants (Basel). 2019 Sep 1;8(9):353. doi: 10.3390/antiox8090353 (PMC6769514; doi:10.3390/antiox8090353)
Supplement: Supplementary file 1 [file antioxidants-08-00353-s001.zip › Table S3.docx]

**Table S3. Terms occurrences and averaged citations from 2011–2015**

| **terms** | **occurrences** | **averaged citations** |
| --- | --- | --- |
| oxidative stress | 2125 | 27.2 |
| activation | 814 | 25.8 |
| pathway | 798 | 27.5 |
| heme oxygenase-1 | 787 | 27.5 |
| expression | 756 | 24.5 |
| gene expression | 691 | 28.9 |
| nf-kappa b | 627 | 27.2 |
| induction | 603 | 26.6 |
| mouse | 483 | 28.0 |
| apoptosis | 433 | 23.0 |
| inflammation | 429 | 28.0 |
| cells | 397 | 23.3 |
| antioxidant | 388 | 24.6 |
| mechanism | 369 | 25.7 |
| reactive oxygen species | 361 | 28.8 |
| transcription factor | 278 | 31.2 |
| keap1 | 272 | 38.8 |
| in-vitro | 270 | 25.5 |
| in-vivo | 269 | 30.1 |
| cancer | 267 | 29.9 |
| antioxidant response element | 258 | 31.6 |
| gene | 252 | 25.3 |
| glutathione | 238 | 24.4 |
| rat | 221 | 22.8 |
| protection | 220 | 25.6 |
| protein | 214 | 28.3 |
| stress | 201 | 25.2 |
| antioxidant response | 192 | 35.7 |
| disease | 187 | 29.7 |
| nitric oxide | 176 | 27.1 |
| inhibition | 167 | 34.0 |
| molecular-mechanisms | 167 | 28.4 |
| free radicals | 166 | 37.0 |
| injury | 156 | 23.0 |
| autophagy | 148 | 34.1 |
| endothelial-cells | 148 | 31.1 |
| cancer cells | 144 | 27.5 |
| chemoprevention | 142 | 24.9 |
| sulforaphane | 139 | 29.13 |
| parkinson's disease | 133 | 29.3 |
| neuroprotection | 128 | 31.4 |
| damage | 125 | 20.5 |
| alzheimer's disease | 118 | 26.7 |
| cell-death | 106 | 29.6 |
| lipid-peroxidation | 106 | 25.0 |
| ischemia-reperfusion injury | 105 | 28.2 |
| identification | 103 | 26.9 |
| dna-damage | 102 | 28.1 |
| nqo1 | 102 | 24.7 |
| toxicity | 102 | 20.0 |
| induced apoptosis | 101 | 23.8 |
| er stress | 99 | 29.9 |
| degradation | 98 | 32.3 |
| epithelial-cells | 97 | 22.7 |
| nitric-oxide synthase | 95 | 22.7 |
| liver | 92 | 22.5 |
| hydrogen-peroxide | 89 | 21.7 |
| metabolism | 89 | 25.8 |
| neurodegeneration | 89 | 31.2 |
| transcription | 88 | 31.3 |
| resistance | 87 | 24.5 |
| lung-cancer | 85 | 34.0 |
| tnf-alpha | 85 | 24.0 |
| proliferation | 84 | 22.9 |
| model | 82 | 27.0 |
| target | 80 | 28.0 |
| brain | 79 | 24.8 |
| carbon-monoxide | 78 | 25.8 |
| macrophages | 77 | 26.8 |
| carcinogenesis | 76 | 29.4 |
| growth | 76 | 23.8 |
| pc12 cells | 75 | 26.2 |
| phosphorylation | 72 | 32.3 |
| superoxide-dismutase | 72 | 23.3 |
| mitochondria | 71 | 30.5 |
| binding | 70 | 27.9 |
| insulin-resistance | 69 | 32.7 |
| atherosclerosis | 66 | 27.1 |
| death | 66 | 27.6 |
| induced oxidative stress | 66 | 29.9 |
| response element | 65 | 33.1 |
| breast-cancer | 63 | 24.3 |
| p62 | 63 | 37.2 |
| adaptive response | 62 | 27.5 |
| mitochondrial dysfunction | 60 | 27.4 |
| cul3-based e3 ligase | 59 | 52.9 |
| differentiation | 59 | 26.8 |
| protein-kinase-c | 57 | 39.0 |
| smooth-muscle-cells | 57 | 29.9 |
| enzymes | 56 | 23.0 |
| hepatotoxicity | 54 | 21.9 |
| lipopolysaccharide | 54 | 20.0 |
| receptor | 54 | 24.2 |
| resveratrol | 54 | 37.5 |
| cytotoxicity | 53 | 21.9 |
| antioxidant enzymes | 52 | 24.2 |
| hepg2 cells | 52 | 23.8 |
| mutations | 52 | 54.7 |
| cardiovascular-disease | 51 | 36.8 |
| curcumin | 51 | 33.3 |
| obstructive pulmonary-disease | 51 | 31.6 |
| aryl-hydrocarbon receptor | 49 | 29.8 |
| p53 | 49 | 27.7 |
| aging | 48 | 33.8 |
| chemotherapy | 48 | 26.0 |
| high-fat diet | 48 | 42.2 |
| ischemia | 48 | 26.6 |
| acid | 47 | 28.5 |
| unfolded protein response | 47 | 28.2 |
| exposure | 46 | 22.1 |
| kinase | 46 | 22.8 |
| prostate-cancer | 46 | 41.6 |
| transcriptional regulation | 46 | 21.4 |
| antioxidant activity | 45 | 20.1 |
| bardoxolone methyl | 45 | 42.2 |
| lung | 45 | 27.8 |
| quercetin | 45 | 28.4 |
| survival | 45 | 26.4 |
| cerebral-ischemia | 44 | 25.0 |
| dysfunction | 44 | 32.0 |
| astrocytes | 43 | 26.9 |
| flavonoids | 43 | 23.5 |
| life-span | 43 | 43.2 |
| lung injury | 43 | 23.1 |
| messenger-rna | 43 | 26.1 |
| activated protein-kinase | 41 | 32.2 |
| neurotoxicity | 41 | 21.4 |
| neuroinflammation | 40 | 33.4 |
| rat-liver | 40 | 23.7 |
| proteasomal degradation | 39 | 51.5 |
| stroke | 39 | 29.7 |
| cell-survival | 38 | 32.8 |
| multiple-sclerosis | 38 | 44.5 |
| skeletal-muscle | 38 | 30.3 |
| vitamin-e | 38 | 27.7 |
| chemoresistance | 37 | 27.7 |
| polyphenols | 37 | 26.1 |
| tumor-growth | 37 | 40.5 |
| neurons | 36 | 32.4 |
| pathogenesis | 36 | 23.8 |
| prevention | 36 | 20.6 |
| akt | 35 | 20.9 |
| down-regulation | 35 | 33.6 |
| glutathione-s-transferase | 35 | 34.8 |
| obesity | 35 | 28.7 |
