# Supplementary material for: A Bibliometric Review of the Keap1/Nrf2 Pathway and its Related Antioxidant Compounds
Source: Antioxidants (Basel). 2019 Sep 1;8(9):353. doi: 10.3390/antiox8090353 (PMC6769514; doi:10.3390/antiox8090353)
Supplement: Supplementary file 1 [file antioxidants-08-00353-s001.zip › Table S4.docx]

**Table S4. Terms occurrences and averaged citations from 2016–2019**

| **terms** | **occurrences** | **averaged citations** |
| --- | --- | --- |
| oxidative stress | 3338 | 5.7 |
| pathway | 1361 | 5.7 |
| activation | 1295 | 5.1 |
| expression | 1206 | 5.6 |
| induced apoptosis | 1015 | 5.1 |
| inflammation | 925 | 5.4 |
| mechanism | 852 | 5.3 |
| nf kappa b | 827 | 6.1 |
| mouse | 739 | 6.2 |
| heme oxygenase-1 | 694 | 6.0 |
| cells | 675 | 5.4 |
| antioxidant | 640 | 5.2 |
| gene-expression | 564 | 6.9 |
| induction | 553 | 5.3 |
| reactive oxygen species | 511 | 6.0 |
| cancer | 476 | 6.2 |
| inhibition | 424 | 4.7 |
| in-vitro | 416 | 6.4 |
| protection | 414 | 4.9 |
| rat | 396 | 5.4 |
| injury | 349 | 5.1 |
| disease | 335 | 5.5 |
| antioxidant response element | 326 | 6.5 |
| stress | 295 | 4.6 |
| autophagy | 273 | 6.8 |
| damage | 262 | 4.6 |
| keap1 | 260 | 5.5 |
| transcription factor | 253 | 7.4 |
| protein | 249 | 6.3 |
| in-vivo | 248 | 6.9 |
| ischemia-reperfusion injury | 239 | 5.7 |
| glutathione | 225 | 4.9 |
| gene | 220 | 7.5 |
| toxicity | 212 | 4.5 |
| alzheimer's disease | 204 | 6.9 |
| metabolism | 195 | 5.1 |
| nf-kappa b | 185 | 6.8 |
| dysfunction | 180 | 4.6 |
| brain | 179 | 4.8 |
| model | 178 | 4.6 |
| nitric-oxide | 177 | 5.6 |
| hepatotoxicity | 168 | 6.8 |
| parkinson's disease | 167 | 6.3 |
| neurodegeneration | 164 | 7.5 |
| neuroprotection | 164 | 5.3 |
| cell-death | 162 | 7.0 |
| endoplasmic reticulum | 158 | 6.1 |
| liver | 158 | 4.0 |
| sulforaphane | 151 | 6.0 |
| dna-damage | 150 | 4.4 |
| lipopolysaccharide | 142 | 5.8 |
| growth | 139 | 5.8 |
| proliferation | 134 | 4.6 |
| endothelial-cells | 132 | 7.0 |
| mitochondria | 131 | 6.5 |
| lipid-peroxidation | 130 | 6.2 |
| resistance | 128 | 5.4 |
| macrophages | 123 | 6.4 |
| insulin-resistance | 122 | 6.2 |
| target | 120 | 4.2 |
| identification | 116 | 6.2 |
| neuroinflammation | 116 | 7.4 |
| mitochondrial dysfunction | 111 | 6.1 |
| acid | 107 | 6.4 |
| system | 106 | 4.9 |
| epithelial-cells | 104 | 5.8 |
| exposure | 104 | 3.0 |
| redox | 100 | 5.2 |
| phosphorylation | 99 | 4.6 |
| death | 97 | 5.9 |
| antioxidant activity | 96 | 4.4 |
| hydrogen-peroxide | 95 | 5.8 |
| pathogenesis | 94 | 3.9 |
| tnf-alpha | 94 | 5.0 |
| therapy | 91 | 5.7 |
| transcription | 91 | 5.9 |
| fibrosis | 90 | 4.3 |
| resveratrol | 90 | 6.1 |
| degradation | 89 | 5.4 |
| receptor | 89 | 4.4 |
| curcumin | 87 | 5.7 |
| survival | 87 | 6.1 |
| differentiation | 85 | 7.4 |
| responses | 83 | 5.3 |
| acute lung injury | 82 | 5.5 |
| extract | 82 | 4.2 |
| nitric-oxide synthase | 80 | 7.5 |
| down-regulation | 79 | 5.1 |
| involvement | 79 | 4.5 |
| nadph oxidase | 78 | 6.8 |
| cytotoxicity | 76 | 6.0 |
| p62 | 75 | 7.2 |
| microglia | 74 | 6.7 |
| modulation | 73 | 5.6 |
| superoxide-dismutase | 73 | 6.0 |
| atherosclerosis | 72 | 5.3 |
| neurotoxicity | 72 | 5.3 |
| oxidative damage | 71 | 6.4 |
| ampk | 70 | 7.4 |
| prevention | 69 | 4.8 |
| diabetes | 68 | 6.1 |
| p53 | 68 | 5.9 |
| lung-cancer | 67 | 5.9 |
| quercetin | 67 | 4.7 |
| antioxidant enzymes | 66 | 5.0 |
| cisplatin | 65 | 4.3 |
| cytokines | 65 | 5.0 |
| kinase | 65 | 7.2 |
| hepg2 cells | 64 | 7.2 |
| neurons | 64 | 4.0 |
| progression | 63 | 5.6 |
| binding | 62 | 7.4 |
| aging | 61 | 5.5 |
| breast-cancer | 61 | 6.2 |
| activated protein-kinase | 60 | 10.9 |
| enzymes | 60 | 5.5 |
| hepatocellular-carcinoma | 60 | 6.6 |
| stroke | 59 | 7.6 |
| liver-injury | 58 | 4.8 |
| pc12 cells | 58 | 6.4 |
| akt | 57 | 6.6 |
| carbon-monoxide | 57 | 5.1 |
| mutations | 57 | 19.0 |
| obesity | 57 | 5.2 |
| unfolded protein response | 57 | 6.6 |
| angiogenesis | 56 | 4.4 |
| carcinogenesis | 56 | 5.0 |
| lung | 56 | 5.1 |
| skeletal-muscle | 55 | 5.3 |
| dimethyl fumarate | 53 | 7.0 |
| high-fat diet | 53 | 6.9 |
| ppar-gamma | 53 | 3.9 |
| flavonoids | 52 | 5.7 |
| hypoxia | 52 | 4.2 |
| lung injury | 52 | 4.6 |
| nephrotoxicity | 52 | 4.5 |
| acute kidney injury | 51 | 6.5 |
| chemotherapy | 51 | 5.9 |
| kidney | 51 | 4.7 |
| polyphenols | 51 | 5.6 |
| life-span | 50 | 9.0 |
| glucose | 49 | 4.9 |
| ischemia | 49 | 4.6 |
| sirt1 | 49 | 6.0 |
| sepsis | 48 | 3.5 |
| astrocytes | 46 | 6.0 |
| cul3-based e3 ligase | 46 | 8.3 |
| hyperglycemia | 46 | 4.7 |
| macular degeneration | 46 | 8.1 |
| mapk | 46 | 5.5 |
| multiple-sclerosis | 46 | 11.5 |
| cerebral-ischemia | 45 | 6.2 |
| diabetic nephropathy | 45 | 6.1 |
| nonalcoholic steatohepatitis | 45 | 5.5 |
