# Supplementary material for: A Bibliometric Review of the Keap1/Nrf2 Pathway and its Related Antioxidant Compounds
Source: Antioxidants (Basel). 2019 Sep 1;8(9):353. doi: 10.3390/antiox8090353 (PMC6769514; doi:10.3390/antiox8090353)
Supplement: Supplementary file 1 [file antioxidants-08-00353-s001.zip › Table S5.docx]

**Table S5. Nrf2-related papers by country for the period 1990–2005 (absolute number and % of global Nrf2-related output) and citations received by these papers.**

| **country** | **papers** | **%** | **averaged citations** |
| --- | --- | --- | --- |
| USA | 208 | 59.9 | 194.7 |
| JAPAN | 103 | 29.7 | 254.6 |
| UK | 38 | 11.0 | 155.8 |
| SOUTH KOREA | 20 | 5.8 | 98.4 |
| GERMANY | 12 | 3.5 | 85.0 |
| CANADA | 9 | 2.6 | 175.0 |
| ITALY | 9 | 2.6 | 99.1 |
| FRANCE | 8 | 2.3 | 102.6 |
| SINGAPORE | 4 | 1.2 | 69.3 |
| SWEDEN | 4 | 1.2 | 30.3 |
| SWITZERLAND | 4 | 1.2 | 245.0 |
| CHINA | 3 | 0.9 | 250.7 |
| SPAIN | 3 | 0.9 | 193.7 |
| AUSTRALIA | 2 | 0.6 | 34.0 |
| AUSTRIA | 2 | 0.6 | 155.5 |
| NORWAY | 2 | 0.6 | 69.5 |
| POLAND | 2 | 0.6 | 23.0 |
| TAIWAN | 2 | 0.6 | 46.0 |
| URUGUAY | 2 | 0.6 | 72.5 |
| ARGENTINA | 1 | 0.3 | 33.0 |
| BELGIUM | 1 | 0.3 | 63.0 |
| ISRAEL | 1 | 0.3 | 185.0 |
| PORTUGAL | 1 | 0.3 | 14.0 |
| RUSSIA | 1 | 0.3 | 127.0 |
| TRINID TOBAGO | 1 | 0.3 | 131.0 |
| VENEZUELA | 1 | 0.3 | 67.0 |

Each paper may be counted by more than one country (international collaboration).
