# Supplementary material for: A Bibliometric Review of the Keap1/Nrf2 Pathway and its Related Antioxidant Compounds
Source: Antioxidants (Basel). 2019 Sep 1;8(9):353. doi: 10.3390/antiox8090353 (PMC6769514; doi:10.3390/antiox8090353)
Supplement: Supplementary file 1 [file antioxidants-08-00353-s001.zip › Table S6.docx]

**Table S6. Nrf2-related papers by country for the period 2006–2010 (absolute number and % of global Nrf2-related output) and citations received by these papers.**

| **country** | **papers** | **%** | **averaged citations** |
| --- | --- | --- | --- |
| USA | 607 | 47.8 | 80.2 |
| JAPAN | 238 | 18.8 | 77.3 |
| SOUTH KOREA | 155 | 12.2 | 46.4 |
| CHINA | 111 | 8.7 | 46.1 |
| UK | 76 | 6.0 | 96.9 |
| GERMANY | 65 | 5.1 | 69.9 |
| ITALY | 39 | 3.1 | 60.2 |
| CANADA | 36 | 2.8 | 66.1 |
| TAIWAN | 32 | 2.5 | 53.8 |
| SPAIN | 31 | 2.4 | 71.0 |
| FRANCE | 30 | 2.4 | 57.6 |
| SWITZERLAND | 23 | 1.8 | 83.8 |
| NETHERLANDS | 18 | 1.4 | 91.3 |
| FINLAND | 17 | 1.3 | 63.3 |
| INDIA | 17 | 1.3 | 92.6 |
| SWEDEN | 12 | 0.9 | 62.3 |
| AUSTRIA | 11 | 0.9 | 41.2 |
| SINGAPORE | 10 | 0.8 | 57.2 |
| POLAND | 9 | 0.7 | 54.0 |
| AUSTRALIA | 8 | 0.6 | 61.9 |
| MEXICO | 8 | 0.6 | 40.9 |
| BELGIUM | 7 | 0.6 | 46.0 |
| THAILAND | 7 | 0.6 | 31.7 |
| IRAN | 6 | 0.5 | 39.0 |
| NORWAY | 6 | 0.5 | 156.5 |
| TURKEY | 6 | 0.5 | 105.0 |
| ISRAEL | 5 | 0.4 | 43.2 |
| GREECE | 5 | 0.4 | 40.8 |
| RUSSIA | 5 | 0.4 | 9.8 |
| NEW ZEALAND | 4 | 0.3 | 45.5 |
| BRAZIL | 4 | 0.3 | 13.0 |
| CZECH REPUBLIC | 3 | 0.2 | 129.7 |
| URUGUAY | 3 | 0.2 | 93.7 |
| IRELAND | 3 | 0.2 | 68.7 |
| MALAYSIA | 3 | 0.2 | 67.7 |
| CHILE | 3 | 0.2 | 59.3 |
| DENMARK | 3 | 0.2 | 49.7 |
| SOUTH AFRICA | 1 | 0.1 | 94.0 |
| ARGENTINA | 1 | 0.1 | 87.0 |
| ESTONIA | 1 | 0.1 | 43.0 |
| SLOVAKIA | 1 | 0.1 | 37.0 |
| PHILIPPINES | 1 | 0.1 | 32.0 |
| VIETNAM | 1 | 0.1 | 24.0 |
| CROATIA | 1 | 0.1 | 20.0 |
| EGYPT | 1 | 0.1 | 8.0 |
| MONGOL PEOP REP | 1 | 0.1 | 6.0 |
| NIGERIA | 1 | 0.1 | 2.0 |

Each paper may be counted by more than one country (international collaboration).
