# Supplementary material for: A Bibliometric Review of the Keap1/Nrf2 Pathway and its Related Antioxidant Compounds
Source: Antioxidants (Basel). 2019 Sep 1;8(9):353. doi: 10.3390/antiox8090353 (PMC6769514; doi:10.3390/antiox8090353)
Supplement: Supplementary file 1 [file antioxidants-08-00353-s001.zip › Table S7.docx]

**Table S7. Nrf2-related papers by country for the period 2011-2015 (absolute number and % of global Nrf2-related output) and citations received by these papers.**

| **country** | **papers** | **%** | **averaged citations** |
| --- | --- | --- | --- |
| USA | 1373 | 33.5 | 1370.0 |
| CHINA | 1063 | 25.9 | 1063.0 |
| SOUTH KOREA | 446 | 10.9 | 446.0 |
| JAPAN | 410 | 10.0 | 410.0 |
| UK | 246 | 6.0 | 227.0 |
| GERMANY | 220 | 5.4 | 35.3 |
| TAIWAN | 161 | 3.9 | 26.2 |
| ITALY | 139 | 3.4 | 30.7 |
| INDIA | 127 | 3.1 | 28.2 |
| SPAIN | 126 | 3.1 | 32.2 |
| CANADA | 105 | 2.6 | 27.4 |
| FRANCE | 79 | 1.9 | 31.0 |
| NETHERLANDS | 59 | 1.4 | 40.3 |
| AUSTRALIA | 53 | 1.3 | 29.0 |
| SWITZERLAND | 51 | 1.2 | 40.1 |
| BRAZIL | 50 | 1.2 | 22.1 |
| AUSTRIA | 45 | 1.1 | 48.6 |
| MEXICO | 40 | 1.0 | 25.3 |
| POLAND | 40 | 1.0 | 18.4 |
| SWEDEN | 33 | 0.8 | 29.3 |
| FINLAND | 32 | 0.8 | 25.9 |
| TURKEY | 32 | 0.8 | 22.5 |
| IRAN | 30 | 0.7 | 20.2 |
| EGYPT | 29 | 0.7 | 17.7 |
| SINGAPORE | 27 | 0.7 | 31.2 |
| ISRAEL | 26 | 0.6 | 26.2 |
| BELGIUM | 23 | 0.6 | 52.1 |
| RUSSIA | 21 | 0.5 | 19.3 |
| NORWAY | 19 | 0.5 | 33.9 |
| DENMARK | 18 | 0.4 | 44.1 |
| PORTUGAL | 18 | 0.4 | 31.9 |
| THAILAND | 17 | 0.4 | 18.3 |
| CHILE | 16 | 0.4 | 14.9 |
| SAUDI ARABIA | 16 | 0.4 | 15.6 |
| GREECE | 15 | 0.4 | 35.3 |
| MALAYSIA | 14 | 0.3 | 13.7 |
| CZECH REPUBLIC | 13 | 0.3 | 21.5 |
| HUNGARY | 13 | 0.3 | 45.2 |
| ARGENTINA | 12 | 0.3 | 13.6 |
| IRELAND | 9 | 0.2 | 34.4 |
| NEW ZEALAND | 7 | 0.2 | 16.9 |
| SOUTH AFRICA | 7 | 0.2 | 13.5 |
| CROATIA | 6 | 0.1 | 20.8 |
| LUXEMBOURG | 6 | 0.1 | 76.7 |
| SERBIA | 6 | 0.1 | 18.0 |
| VIETNAM | 6 | 0.1 | 8.7 |
| NIGERIA | 5 | 0.1 | 20.6 |
| ROMANIA | 5 | 0.1 | 12.2 |
| COLOMBIA | 4 | 0.1 | 10.5 |
| SLOVAKIA | 3 | 0.1 | 31.0 |
| URUGUAY | 3 | 0.1 | 27.7 |
| CAMEROON | 2 | 0.04 | 8.0 |
| INDONESIA | 2 | 0.05 | 40.0 |
| IRAQ | 2 | 0.05 | 31.5 |
| KUWAIT | 2 | 0.05 | 32.0 |
| PAKISTAN | 2 | 0.05 | 41.5 |
| TUNISIA | 2 | 0.05 | 21.0 |
| UKRAINE | 2 | 0.05 | 1.0 |
| VENEZUELA | 2 | 0.05 | 43.0 |
| BANGLADESH | 1 | 0.02 | 0 |
| BENIN | 1 | 0.02 | 3.0 |
| CYPRUS | 1 | 0.02 | 10.0 |
| ESTONIA | 1 | 0.02 | 56.0 |
| ETHIOPIA | 1 | 0.02 | 7.0 |
| KENYA | 1 | 0.02 | 12.0 |
| LIBYA | 1 | 0.02 | 23.0 |
| MALTA | 1 | 0.02 | 28.0 |
| MONACO | 1 | 0.02 | 5.0 |
| OMAN | 1 | 0.02 | 7.0 |
| QATAR | 1 | 0.02 | 8.0 |
| SUDAN | 1 | 0.02 | 23.0 |
| SYRIA | 1 | 0.02 | 23.0 |

Each paper may be counted by more than one country (international collaboration).
