# Supplementary material for: A Bibliometric Review of the Keap1/Nrf2 Pathway and its Related Antioxidant Compounds
Source: Antioxidants (Basel). 2019 Sep 1;8(9):353. doi: 10.3390/antiox8090353 (PMC6769514; doi:10.3390/antiox8090353)
Supplement: Supplementary file 1 [file antioxidants-08-00353-s001.zip › Table S8.docx]

**Table S8. Nrf2-related papers by country for the period 2016–2019 (absolute number and % of global Nrf2-related output) and citations received by these papers.**

| **country** | **papers** | **%** | **averaged citations** |
| --- | --- | --- | --- |
| CHINA | 2815 | 45.3 | 5.4 |
| USA | 1279 | 20.6 | 8.1 |
| SOUTH KOREA | 545 | 8.8 | 5.4 |
| JAPAN | 391 | 6.3 | 6.6 |
| GERMANY | 237 | 3.8 | 7.8 |
| INDIA | 230 | 3.7 | 5.3 |
| UK | 221 | 3.6 | 8.8 |
| ITALY | 219 | 3.5 | 6.8 |
| SPAIN | 170 | 2.7 | 7.9 |
| BRAZIL | 167 | 2.7 | 5.0 |
| TAIWAN | 164 | 2.6 | 4.8 |
| EGYPT | 154 | 2.5 | 4.3 |
| CANADA | 121 | 1.9 | 6.1 |
| FRANCE | 107 | 1.7 | 6.0 |
| AUSTRALIA | 78 | 1.3 | 6.8 |
| SAUDI ARABIA | 69 | 1.1 | 5.0 |
| IRAN | 63 | 1.0 | 5.0 |
| SWITZERLAND | 60 | 1.0 | 11.8 |
| NETHERLANDS | 58 | 0.9 | 6.6 |
| MEXICO | 55 | 0.9 | 5.1 |
| SWEDEN | 53 | 0.9 | 6.9 |
| POLAND | 51 | 0.8 | 5.5 |
| TURKEY | 49 | 0.8 | 5.1 |
| THAILAND | 44 | 0.7 | 7.7 |
| RUSSIA | 41 | 0.7 | 4.5 |
| FINLAND | 36 | 0.6 | 7.3 |
| AUSTRIA | 34 | 0.5 | 7.7 |
| SINGAPORE | 28 | 0.5 | 7.8 |
| ISRAEL | 27 | 0.4 | 4.6 |
| PORTUGAL | 24 | 0.4 | 8.7 |
| DENMARK | 23 | 0.4 | 6.1 |
| ARGENTINA | 22 | 0.4 | 5.3 |
| MALAYSIA | 22 | 0.4 | 4.9 |
| BELGIUM | 21 | 0.3 | 12.6 |
| CHILE | 20 | 0.3 | 4.7 |
| NORWAY | 20 | 0.3 | 5.7 |
| PAKISTAN | 20 | 0.3 | 4.4 |
| GREECE | 19 | 0.3 | 4.4 |
| HUNGARY | 19 | 0.3 | 2.9 |
| CZECH REPUBLIC | 18 | 0.3 | 6.1 |
| SOUTH AFRICA | 18 | 0.3 | 6.4 |
| NIGERIA | 16 | 0.3 | 2.6 |
| U ARAB EMIRATES | 15 | 0.2 | 4.2 |
| ROMANIA | 12 | 0.2 | 4.2 |
| VIETNAM | 11 | 0.2 | 5.6 |
| IRELAND | 10 | 0.2 | 13.9 |
| SERBIA | 9 | 0.1 | 3.9 |
| CROATIA | 8 | 0.1 | 4.1 |
| INDONESIA | 8 | 0.1 | 1.0 |
| LUXEMBOURG | 8 | 0.1 | 11.9 |
| SLOVAKIA | 8 | 0.1 | 3.8 |
| OMAN | 7 | 0.1 | 3.5 |
| URUGUAY | 7 | 0.1 | 4.3 |
| QATAR | 6 | 0.1 | 7.7 |
| UKRAINE | 5 | 0.1 | 1.4 |
| BANGLADESH | 4 | 0.1 | 2.8 |
| COLOMBIA | 4 | 0.1 | 4.5 |
| JORDAN | 4 | 0.1 | 1.5 |
| NEW ZEALAND | 4 | 0.1 | 0 |
| TUNISIA | 4 | 0.1 | 1.3 |
| BULGARIA | 3 | 0.05 | 2.0 |
| ESTONIA | 3 | 0.05 | 1.0 |
| LEBANON | 3 | 0.05 | 7.5 |
| ALGERIA | 2 | 0.03 | 13.0 |
| ECUADOR | 2 | 0.03 | 46.5 |
| ETHIOPIA | 2 | 0.03 | 0 |
| IRAQ | 2 | 0.03 | 0 |
| LATVIA | 2 | 0.03 | 2.0 |
| MALTA | 2 | 0.03 | 13.5 |
| SLOVENIA | 2 | 0.03 | 0.5 |
| SRI LANKA | 2 | 0.03 | 3.0 |
| SUDAN | 2 | 0.03 | 5.0 |
| MONGOL PEO REP | 2 | 0.03 | 2.5 |
| AFGHANISTAN | 1 | 0.02 | 1.0 |
| BARBADOS | 1 | 0.02 | 2.0 |
| BELARUS | 1 | 0.02 | 2.0 |
| CAMBODIA | 1 | 0.02 | 2.0 |
| CAMEROON | 1 | 0.02 | 1.0 |
| CUBA | 1 | 0.02 | 4.0 |
| CYPRUS | 1 | 0.02 | 3.0 |
| ERITREA | 1 | 0.02 | 19.0 |
| FIJI | 1 | 0.02 | 4.0 |
| GHANA | 1 | 0.02 | 1.0 |
| KAZAKHSTAN | 1 | 0.02 | 0 |
| KUWAIT | 1 | 0.02 | 4.0 |
| LIBYA | 1 | 0.02 | 6.0 |
| MOLDOVA | 1 | 0.02 | 12.0 |
| NORTH KOREA | 1 | 0.02 | 0 |
| PANAMA | 1 | 0.02 | 0 |
| PHILIPPINES | 1 | 0.02 | 2.0 |
| UGANDA | 1 | 0.02 | 0 |
| UZBEKISTAN | 1 | 0.02 | 5.0 |

Each paper may be counted by more than one country (international collaboration).
