# Supplementary material for: A Bibliometric Review of the Keap1/Nrf2 Pathway and its Related Antioxidant Compounds
Source: Antioxidants (Basel). 2019 Sep 1;8(9):353. doi: 10.3390/antiox8090353 (PMC6769514; doi:10.3390/antiox8090353)
Supplement: Supplementary file 1 [file antioxidants-08-00353-s001.zip › Table S9.docx]

**Table S9. Nrf2-related papers by country for the period 1990—2019 (absolute number and % of global Nrf2-related output) and citations received by these papers.**

| **country** | **papers** | **%** | **averaged citations** |
| --- | --- | --- | --- |
| CHINA | 3992 | 33.5 | 12.1 |
| USA | 3467 | 29.1 | 42.6 |
| SOUTH KOREA | 1166 | 9.8 | 19.7 |
| JAPAN | 1142 | 9.6 | 53.5 |
| UK | 581 | 4.9 | 43.8 |
| GERMANY | 534 | 4.5 | 28.6 |
| ITALY | 406 | 3.4 | 22.5 |
| INDIA | 374 | 3.1 | 17.5 |
| TAIWAN | 359 | 3.0 | 19.4 |
| SPAIN | 330 | 2.8 | 25.3 |
| CANADA | 271 | 2.3 | 28.3 |
| FRANCE | 224 | 1.9 | 25.5 |
| BRAZIL | 221 | 1.9 | 9.1 |
| EGYPT | 184 | 1.5 | 6.6 |
| AUSTRALIA | 141 | 1.2 | 19.0 |
| SWITZERLAND | 137 | 1.1 | 41.2 |
| NETHERLANDS | 135 | 1.1 | 33.0 |
| MEXICO | 103 | 0.9 | 15.7 |
| POLAND | 102 | 0.9 | 15.2 |
| SWEDEN | 102 | 0.9 | 22.0 |
| IRAN | 99 | 0.8 | 12.0 |
| AUSTRIA | 92 | 0.8 | 34.9 |
| TURKEY | 87 | 0.7 | 18.4 |
| FINLAND | 85 | 0.7 | 25.7 |
| SAUDI ARABIA | 85 | 0.7 | 7.2 |
| SINGAPORE | 69 | 0.6 | 28.3 |
| RUSSIA | 68 | 0.6 | 11.7 |
| THAILAND | 68 | 0.6 | 12.9 |
| ISRAEL | 59 | 0.5 | 20.7 |
| BELGIUM | 52 | 0.4 | 35.5 |
| NORWAY | 47 | 0.4 | 39.8 |
| DENMARK | 44 | 0.4 | 24.6 |
| PORTUGAL | 43 | 0.4 | 18.8 |
| CHILE | 39 | 0.3 | 13.1 |
| GREECE | 39 | 0.3 | 21.8 |
| MALAYSIA | 39 | 0.3 | 12.9 |
| ARGENTINA | 36 | 0.3 | 11.3 |
| CZECH REPUBLIC | 34 | 0.3 | 23.4 |
| HUNGARY | 32 | 0.3 | 20.1 |
| SOUTH AFRICA | 28 | 0.2 | 11.8 |
| IRELAND | 22 | 0.2 | 29.8 |
| NIGERIA | 22 | 0.2 | 7.9 |
| PAKISTAN | 22 | 0.2 | 8.0 |
| VIETNAM | 18 | 0.2 | 8.7 |
| ROMANIA | 17 | 0.1 | 6.7 |
| CROATIA | 15 | 0.1 | 11.9 |
| NEW ZEALAND | 15 | 0.1 | 21.6 |
| SERBIA | 15 | 0.1 | 9.3 |
| U ARAB EMIRATES | 15 | 0.1 | 4.2 |
| URUGUAY | 15 | 0.1 | 35.9 |
| LUXEMBOURG | 14 | 0.1 | 41.8 |
| SLOVAKIA | 12 | 0.1 | 16.6 |
| INDONESIA | 10 | 0.1 | 9.7 |
| COLOMBIA | 8 | 0.1 | 7.5 |
| OMAN | 8 | 0.1 | 4.0 |
| QATAR | 7 | 0.1 | 7.7 |
| UKRAINE | 7 | 0.1 | 1.3 |
| TUNISIA | 6 | 0.1 | 7.8 |
| BANGLADESH | 5 | 0.04 | 2.8 |
| ESTONIA | 5 | 0.04 | 25.3 |
| IRAQ | 4 | 0.03 | 21.0 |
| JORDAN | 4 | 0.03 | 1.5 |
| BULGARIA | 3 | 0.03 | 2.0 |
| CAMEROON | 3 | 0.03 | 5.7 |
| ETHIOPIA | 3 | 0.03 | 2.3 |
| KUWAIT | 3 | 0.03 | 22.7 |
| LEBANON | 3 | 0.03 | 7.5 |
| MALTA | 3 | 0.03 | 18.3 |
| MONGOL PEO REP | 3 | 0.03 | 3.7 |
| SUDAN | 3 | 0.03 | 11.0 |
| VENEZUELA | 3 | 0.03 | 51.0 |
| ALGERIA | 2 | 0.02 | 13.0 |
| CYPRUS | 2 | 0.02 | 6.5 |
| ECUADOR | 2 | 0.02 | 46.5 |
| LATVIA | 2 | 0.02 | 2.0 |
| LIBYA | 2 | 0.02 | 14.5 |
| PHILIPPINES | 2 | 0.02 | 17.0 |
| SLOVENIA | 2 | 0.02 | 0.5 |
| SRI LANKA | 2 | 0.02 | 3.0 |
| AFGHANISTAN | 1 | 0.01 | 1.0 |
| BARBADOS | 1 | 0.01 | 2.0 |
| BELARUS | 1 | 0.01 | 2.0 |
| BENIN | 1 | 0.01 | 3.0 |
| CAMBODIA | 1 | 0.01 | 2.0 |
| CUBA | 1 | 0.01 | 4.0 |
| ERITREA | 1 | 0.01 | 19.0 |
| FIJI | 1 | 0.01 | 4.0 |
| GHANA | 1 | 0.01 | 1.0 |
| KAZAKHSTAN | 1 | 0.01 | 0 |
| KENYA | 1 | 0.01 | 12.0 |
| MOLDOVA | 1 | 0.01 | 12.0 |
| MONACO | 1 | 0.01 | 5.0 |
| NORTH KOREA | 1 | 0.01 | 0 |
| SYRIA | 1 | 0.01 | 23.0 |
| TRINID TOBAGO | 1 | 0.01 | 131.0 |
| UGANDA | 1 | 0.01 | 0 |
| UZBEKISTAN | 1 | 0.01 | 5.0 |

Each paper may be counted by more than one country (international collaboration).
